# Using Project Performance to Measure Effectiveness of Quality Management System Maintenance and Practices in Construction Industry

**DOI:** 10.1155/2014/591361

**Published:** 2014-02-19

**Authors:** Tiong Kung Leong, Norhayati Zakuan, Muhamad Zameri Mat Saman, Mohd. Shoki Md. Ariff, Choy Soon Tan

**Affiliations:** ^1^Faculty of Management, Universiti Teknologi Malaysia, 81310 Skudai, Johor, Malaysia; ^2^Faculty of Mechanical Engineering, Universiti Teknologi Malaysia, 81310 Skudai, Johor, Malaysia; ^3^Faculty of Civil Engineering, Universiti Teknologi Malaysia, 81310 Skudai, Johor, Malaysia

## Abstract

This paper proposed seven existing and new performance indicators to measure the effectiveness of quality management system (QMS) maintenance and practices in construction industry. This research is carried out with a questionnaire based on QMS variables which are extracted from literature review and project performance indicators which are established from project management's theory. Data collected was analyzed using correlation and regression analysis. The findings indicate that client satisfaction and time variance have positive and significant relationship with QMS while other project performance indicators do not show significant results. Further studies can use the same project performance indicators to study the effectiveness of QMS in different sampling area to improve the generalizability of the findings.

## 1. Introduction

Quality management system has been widely implemented and adopted in construction industry especially those companies which are able to handle megaprojects. Although uncountable researches have been carried out to study the relationship of quality management system with various industries (e.g., manufacturing, food, service, etc.), there is lack of relevant studies on construction industry. It is because those researchers are more interested in looking into the quality of the project works and costing of the projects in construction industry [1–3] compared to quality management system. Those studies conducted some international comparisons on construction quality among United States, United Kingdom, and Japan [2] and between Singapore and Hong Kong [3]. Some robust benchmarks have been generated through these attempts for the professionals in various countries so that they can improve their competitiveness.

Furthermore, some studies have examined the effects and benefit of implementing quality management system in construction industry. Some evidence finds that implementing quality management system can improve communication problems; minimize mistakes, rework, and material wastage; have better control of subcontractors and suppliers. Hence, the productivity, profitability, and market share have been generally increased and also enable contractors to meet the client requirements [4]. Besides, there is a study trying to construct a model for the quality management system maintenance processes because it has been suggested as an essential activity for every organization including construction industry [5]. Initially, maintenance concepts have been linked with concept of quality management matrix to define the maintenance of quality management [6–8].

Nonetheless, most of the quality management system studies are not specifically designed to examine the objectives (time, cost, and quality) in construction industry and usually the scope of study will include numerous industries for comparison purpose. For example, there is a study that identified 10 critical success factors for quality management system implementation after reviewing 100 studies from different industries backgrounds [9]. Furthermore, some researchers have focused their studies on relationship between quality management system and organizational performance and business performance instead of project performance [10, 11]. Some studies also indicated that implementation of ISO 9000 can benefit organizations by improving production performance and quality awareness of the employees in diverse industries [12–16].

Even though there are many studies indicating that some organizations have received benefits of quality and also increased their productivity after implementing quality management system, there are limited researches looking into the relationship between QMS and project performance. Therefore, there is a need to know whether QMS is able to improve project performance so that the benefit and effectiveness of QMS can be measured with project management theory. Furthermore, some new project performance indicators will be used in this research to establish significant performance indicators.

## 2. Literature Review

Effectiveness and efficiency of quality management implementation are important for improving performance of an organization. In the past quality management literature, there are many studies investigating the effects of different quality management practices on quality performance [17–20], operational performance [21–23], and business performance [24, 25]. Most of the findings have indicated that quality management practices have a clear and significant relationship with quality and operational performance, but they have insignificant and unclear effects on business performance. Furthermore, a research shows that quality can assist a company to obtain competitive advantages by meeting customer needs when delivering quality products to the marketplace. The results also indicate that the quality dimensions which have been used in the research are correlated with business performance [26]. Nevertheless, those performance indicators are not suitable for measuring construction projects.

The success of a construction project can be indicated by project performance. The performance of a project will be dependent on various factors including project complexity, contractual arrangements, relationships between participants in the project, competency of project manager, and the abilities of the key members in projects. Project performance is usually judged and quantified by performance measurement. Performance measurement is the common method to collect and report the information related to the inputs, efficiency, and effectiveness of a construction project [27]. Furthermore, measurements are crucial for tracking, forecasting, and controlling the important variables in the end to ensure the success of projects [28–32].

**Figure 1:**
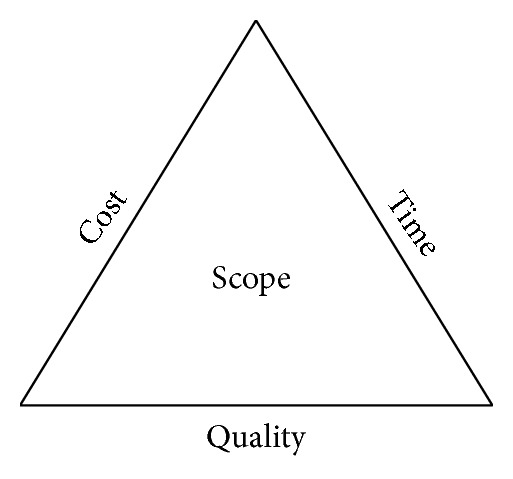
The iron triangle of project management.

Generally, it has been widely accepted that time, cost, and quality are the major concerned factors in the performance measurements of a project [33]. Moreover, these three major components are noted as “iron triangle” (see Figure 1) according to a researcher [34]. On the other hand, there are other criteria that have been suggested to be considered in a project [35]. Those criteria include meeting the budget, schedule, quality of workmanship, stakeholders' satisfaction, safety and health, and transfer of technology. At the same time, some researchers also noted that there are other various key components such as design performance, safety and health, performance of environmental management, expectation or satisfaction of the end user, client's satisfaction, and commercial value that are used in project performance measurement [36]. Hence, there are five major variables that have been identified to measure projects performance. There are cost performance, time performance, quality performance, safety and health, and clients' satisfaction.

### 2.1. Cost Performance

It is stated as the degree to which the general conditions promote the completion of a project within the estimated budget [37]. The cost variance is the technique usually used for design performance measurement of a project in construction industry [38]. Moreover, cost variance technique is not only limited to the calculation of the tender sum but also includes the overall costing that incurs in a project from commencement to completion. The overall costs comprise all the costs involved in variation work or modification work and also the costs involved in any legal claims such as arbitration or litigation upon the construction period. Cost variance is measured in terms of unit cost, percentage of net variation over the final cost [36]. Besides, Cost variance has been used in a research as the measurement for project performance in a construction project which has problems of defective design [39]. Furthermore, the element of cost also has been suggested to measure the performance in engineering projects [40]. Besides cost variance, cost performance index (CPI) also has been used to measure performance of a project for the reliability and the confidence of results. The formula of the elements and indication are shown as follows.

#### 2.1.1. Cost Variance (CV)

The project is ideally on budget when the value of CV is equal to zero. When the CV value is greater than zero, that means the earning of project has more value than the planned earning; therefore it is under budget. When the CV value is less than zero, that means the earning of project has less value than the planned earning; hence it is over budget

(1) $$\begin{array}{c} \text{CV}=\text{BCWP}-\text{ACWP}, \end{array}$$

where BCWP is the budgeted cost of work performed and ACWP is the actual cost of work performed.

#### 2.1.2. Cost Performance Index (CPI)

The project is ideally on budget when the value of CPI is equal to one. When the CPI value is less than one, that means the project is over budget. When the CPI value is greater than one, that means the project is under budget. A project with good performance must maintain its CPI value as near to one as possible

(2) $$\begin{array}{c} \text{CPI}=\frac{\text{BCWP}}{\text{ACWP}}. \end{array}$$

### 2.2. Time Performance

From the perspective of client, end users, stakeholders, or the general public, the first criteria to measure success of the project will be the completion time. Therefore, it is very crucial to complete the construction project on time when people judge the project success from the macroview [41]. Time variance (TV) has been suggested as one of the techniques of assessing performance of project in construction industry [38, 42]. The formula for time variance is shown as below:

(3) $$\begin{array}{c} \text{TV}=\text{BTWP}-\text{ATWP}, \end{array}$$

where BTWP is the budgeted time of work performed and ATWP is the actual time of work performed.

The indication from the element of time can give awareness for project manager to be aware that the project is not running as well as scheduled. Moreover, delivery of projects on time has been suggested as one of the main requirements of clients in the construction contracts [43].

### 2.3. Quality Performance

Quality is described as the totality of features required by a product or service to satisfy a given need; fitness for purpose [44]. In other words, quality in construction industry emphasizes the capability to establish requirements with conformance to the quality standard. Requirements will be predefined by client in contract agreement and the requirements consist of the established characteristics of products, processes, and services. All the parties involved in the project must fully understand those requirements and expectation in order to achieve a complete project that meets clients' quality expectation [45]. Quality performance can be measured by looking into the nonconformance report (NCR) in the ISO 9000 certified company. Moreover, quality performance can be determined by taking clients' satisfaction into consideration.

### 2.4. Client's Satisfaction

Satisfaction has been explained as a function to make comparison between a perception of an outcome by an individual and the expectation of the outcome [46]. Client's satisfaction has become challenging issue for the past few decades in construction industry. Usually, clients of construction sector experienced dissatisfaction in many aspects including overspend in project cost, delay of completion, poor quality, and incompetent project teams like subcontractors and consultants. There is a research suggesting that to build up relationship with a new client in construction industry is five times more expensive than to maintain existing one [47]. The findings also show that construction companies can increase their profits by 100 percent if they are able to retain five percent or more from the existing clients.Therefore, clients' satisfaction is one of the key performance indicators for all the participants in construction industry. They must always show improvement in the performance if they want to survive and sustain in the global marketplace. However, quality performance of the products and services which have been received by client within cost and time is always tightly related to the measurement of clients' satisfaction [48, 49].

### 2.5. Safety and Health

Safety and health is stated as the degree to which the general conditions promote the completion of a project without major fatalities or injuries [37]. Measurement of safety is primarily look at the amount of accidents occur during construction period. Construction works are well known as one of the most dangerous and risky activities throughout the world because large amount of people are being killed and injured every year. The finding of a research indicates that construction worker has three times more higher chances of dying and two times higher chances of getting injured compared to the workers involved in other industrial activities [50]. The safety performance is traditionally measured by using statistic data of injury and fatality. The main intention in measuring safety and health performance is providing information on the progress and current situation of site activities to control safety and health risks. Moreover, measurement will be only considered effective when it is reporting on the risk levels and also investigating the reason of exposing to current risk level. Then, the corrective actions will be taken to improve overall project performance ultimately.

## 3. Research Methodology

This study conducted survey to obtain the quantitative and qualitative profile of the QMS maintenance and practices towards project performance in construction industry. The survey is carried out by using questionnaires. The questionnaires are delivered by researchers. This method is employed in the study because face-to-face survey interview can clarify the questions, clear doubts, and make sure the questions are fully understood by the respondents before they answer the questionnaires. Besides, this method will reduce the “nonresponse” rate and obtain richer data about the QMS maintenance during the interview session. The following sections provide a detailed description of the methodology utilized in the survey.

### 3.1. Population

The population of this research consists of all the local construction projects of construction companies in Malaysia. All the constructions projects in Malaysia have been recorded by Construction Industry Development Board (CIDB) and statistics data are presented in Construction Quarterly Statistical Bulletin [51]. The total of the construction projects is 7,359 for the year 2011. However, those small scale companies have too little amount of management staffs for each project and normally main contractors will play main role in overall project management. Therefore, only Grade 7 companies' projects will be selected and the total population will be 3,781 for this research.

### 3.2. Research Sample Size and Sample Selection

Sampling is necessary in this research because the population is large and widely spread in every state of Malaysia. Sample comprises selected construction projects at every state of Malaysia from the population. The calculation for the minimum sample in this research is based on Cochran's formula [52]. Based on the calculation, minimum sample is 349 construction projects. Each sample will provide 3 respondents including supervisors, administrators, engineers, quantity surveyors, and managers which are randomly chosen from project management team for the questionnaire survey. Furthermore, cluster sampling has been selected because it can reduce the cost and time consumed to carry out the research. This type of sampling involves process of area identification, followed by random selection of sample in each area [53].

### 3.3. Questionnaire Design

There are three parts in the questionnaire. Part A consists of the personal details of the respondents which include information of gender, age, highest level of education, position, project involved (completed), and project location. The questions involved in Part B are close-ended and objective questions. These questions measure the implementation of QMS maintenance and practices in the construction companies. Seven-point Likert Scale has been chosen for those objective questions. Part C consists of some structured questions, which gathers the information of the companies and construction project. Besides, those structured questions also contain the project performance measurements by using the project performance indicators (cost variance, cost performance index, time variance, nonconformance reports, client satisfaction, fatality, and number of accidents) which are established from literature review. Both reliability and validity have been checked to make sure the questionnaire is reliable and valid.

### 3.4. Statistical Analysis Techniques Used

The data from the questionnaires will be analyzed by using Statistical Package for Social Sciences (SPSS). Information of part A (demographic factor) and project's information in part C are analyzed by using frequency distribution. At the same time, information from part B is organized and analyzed with information from part A and part C by using correlation and regression analysis.

## 4. Findings

The total number of respondents was 1050 and they are from 350 different construction projects which are scattered in Malaysia. The projects in construction are generally divided into 3 categories which are building, civil, and mechanical and electrical. A breakdown of the number of respondents obtained and the information of the project in which they are involved are detailed in Table 1.

### 4.1. Effectiveness of QMS in Project Performance

Effectiveness of QMS variables in project performance was tested with regression analysis. Project performance was broken down into seven variables including cost variance (CV), cost performance index (CPI), time variance (TV), nonconformance report (NCR), client satisfaction (CS), number of accidents (NA), and fatalities (F). Regression quantified the effectiveness of QMS variables in project performance. At the same time, regression explored pattern of relationship of QMS variables to every project performance indicator. QMS variables illustrate significant positive impact on client satisfaction and time variance. At the same time, QMS variables also show significant negative impact on the number of accidents and fatalities (Table 2).

### 4.2. Relationship between QMS and Project Performance

Correlation determined the relationship of every project performance indicator to QMS variables. QMS variables show significant but weak correlation with client satisfaction and time variance and very weak correlation with other significant project performance indicators (Table 3).

## 5. Discussion

Most of the companies adopt quality management to get competitiveness by improving quality performance [54–56] while construction companies implement QMS to improve their project performance (time, cost, and quality). There are seven indicators that have been used in this research to measure project performance, including cost variance, cost performance index, time variance, nonconformance reports, client satisfaction, number of accidents, and fatalities. Usually contractors will emphasize time, cost, and quality, but none of them can indicate overall project performance.

According to the client structure of a construction company [57], client (developer) will hire and manage the contractors to ensure the quality of the end products before handover to end user. Furthermore, clients will normally list their requirements in detailed descriptions in the contract when awarding the project to contractors. That means client will have checklist and specification to evaluate project management performance from various aspects which have been stated in the contract. Hence, the client satisfaction may represent the overall project performance. In this research, two project performance indicators (client satisfaction and time variance) have shown significant positive relationship with QMS variables in regression analysis. At the same time, both client satisfaction and time variance also illustrated weak but significant correlation with QMS variables. That has explained why time is always tightly related to the measurement of clients' satisfaction in previous studies [48, 49].

On the other hand, there are limited studies that measure project performance by cost variance, cost performance index, number of accidents, and fatalities. From the results of correlation and regression analysis, those project performances do not show significant or positive relationship with QMS variable. For cost variance and cost performance index, the result is not significant maybe due to the following reasons. The construction works are complicated for megaprojects and the works will involve additional cost when there are variation orders (additional works which are instructed by client) from client or rapid changes in design from consultants. Some of the cost will be absorbed by main contractors in the end of projects when the source of the problem cannot be judged.

Meanwhile, Incompetent of nominated subcontractors may be the reason causing the results for number of accidents and fatalities become not significant. Some of the nominated subcontractors' workers are not aware of the safety issues at site especially those foreign general workers. Most of the time, safety and health induction training is not effective to those foreign general workers because they unable to gain the knowledge during safety training due to their poor proficiency of English language. Hence, safety and health induction training becomes standard procedure to fulfill the audit requirement of OHSAS 18000 (an international occupational health and safety management system specification). Nevertheless, nonconformance reports have shown positive but very weak relationship with QMS variables in correlation analysis. The reason may be the size and duration of the project. Megaproject will usually take longer duration to complete, involving more construction activities and subcontractors. All those factors will increase the chances of human errors. Hence, possibility of getting nonconformance reports also increases when the project size is getting bigger or more complicated.

Justification of each type of analysis result and project performance indicators used in this research is crucial for interpretation and further discussion of this research. In this context, In this context, QMS variables are refer to the requirements which have been set out in Clause 4 of the ISO 9000 standards when those ISO certified companies implemented quality management systems [58]. The overall results show that QMS variables have positive and significant relationship with client satisfaction and time variance. That coincides with the finding of an ISO 9000 study in Malaysia construction industry. That research concluded client satisfaction as one of the most important criteria to measure construction project performance [47] even though time and cost have been widely accepted as performance measures of a construction project [33].

Moreover, there are some studies also showing that QMS maintenance and practices can improve customer satisfaction and also quality performance such as structure documentation procedures and better control; cut the cost of project; reduce wastage of project; decrease chance to rework; and diminish conflicts, claims and disputes [59–65]. Nonetheless, those quality performance indicators are not used in this research because there is no standardized formula or measurement tools for those indicators. Furthermore, there is a research indicating that time variance has positive relationship with QMS maintenance and practices [66] which is supported by the result of this research also. For the other five project performance indicators, there are limited researches which are fully focused on the relationship between project performance and QMS maintenance and practices for comparison [67].

Nevertheless, there are some studies stating that some companies may not have improvement on performance after implementation of QMS [68–76]. Thus, QMS variables do not show strong relationship with client satisfaction because the data is only collected from construction companies within Malaysia. However, results of this research conclude that QMS maintenance and practices are generally able to improve project performance and overall performance which have been evidenced by client satisfaction and time variance from this research and other aspects from previous studies.

## 6. Conclusion

This research was set out to examine and verify the relationship between project performance indicators and QMS variables to contribute new knowledge of measuring project performance to construction industry. New and existing project performance indicators are assessed together towards QMS variables for comparison. The findings indicate that client satisfaction and time variance have positive and significant relationship with QMS while other project performance indicators do not show significant results. Previous studies have tended to examine effectiveness of QMS with other performance indicators. Moreover, there are limited researches in construction industry using project performance indicators to measure the effectiveness of QMS maintenance and practices. The findings of this research have enhanced the theory of project performance and QMS.

Nevertheless, a limitation of the research method is that the sampling of research is confined to Malaysia construction industry. Construction companies in different countries may have different results because their managing culture and environment are different. Therefore, that will affect the effectiveness of QMS maintenance and practices in project performance. However, the limitations above are uncertainty for this research because the data collected have passed the reliability and validation test. Thus, the thesis has resulted in new findings and raises further areas that need to be explored. The recommendation to resolve the issues is doing further replications of the study in different countries in order to improve the generalizability of the findings. Further studies can also examine project performance indicators with other types of QMS such as total quality management and Kaizen.

## Figures and Tables

**Table 1:** Profile of respondents.

Factor	Building		Civil		M and E		Overall	
	Freq.	%	Freq.	%	Freq.	%	Freq.	%
Gender								
Male	494	62.8	72	63.2	101	67.3	667	63.5
Female	292	37.2	42	36.8	49	32.7	383	36.5
Age								
20s	350	44.5	55	48.2	69	46.0	474	45.1
30s	399	50.8	52	45.6	72	48.0	523	49.8
40s	26	3.3	7	6.1	7	4.7	40	3.8
50s	8	1.0	0	0.0	2	1.3	10	1.0
60s and above	3	0.4	0	0.0	0	0.0	3	0.3
Role of interviewee's company in project								
Developer	6	0.8	0	0.0	0	0.0	6	0.6
Consultant	3	0.4	3	2.6	0	0.0	6	0.6
Main contractor	744	94.6	108	94.8	117	78.0	969	92.2
Subcontractor	33	4.2	3	2.6	33	22.0	69	6.6
Year of service								
2 years and below	208	26.5	35	30.6	42	28.0	285	27.1
3 to 5 years	420	53.3	57	50.0	70	46.6	547	52.1
6 to 10 years	132	16.8	19	16.7	30	20.0	181	17.2
11 to 15 years	21	2.7	2	1.8	4	2.7	27	2.6
16 to 20 years	3	0.4	1	0.9	4	2.7	8	0.8
21 years and above	2	0.3	0	0.0	0	0.0	2	0.2
Position								
Top management^1^	66	8.4	9	7.9	11	7.3	86	8.2
Middle management^2^	628	79.9	89	78.1	121	80.7	838	79.8
Lower management^3^	92	11.7	16	14.0	18	12.0	126	12.0

^1^Managers level and above. ^2^Engineers, quantity surveyor, contract executive, and quality management executive. ^3^Supervisor, site administrator, and drought person.

**Table 2:** Regression result.

Dependent variable	Model	*B*	Std. error	Sig.
Cost variance	Constant	3.816	0.246	0.000
	QMS variables	−0.059	0.055	0.284

Cost performance index	Constant	3.327	0.097	0.000
	QMS variables	0.004	0.022	0.842

Client satisfaction*	Constant	4.567	0.098	0.000
	QMS variables	0.178	0.022	0.000

Nonconformance report	Constant	5.206	0.184	0.000
	QMS variables	0.065	0.041	0.115

No. of accidents	Constant	5.114	0.044	0.000
	QMS variables	−0.032	0.010	0.301

Time variance*	Constant	3.503	0.221	0.000
	QMS variables	0.345	0.049	0.000

Fatalities	Constant	2.062	0.015	0.000
	QMS variables	−0.016	0.003	0.145

*Significant at 0.05 level.

**Table 3:** Correlation result.

Variable	QMS variables
Cost variance	Not significant
Cost performance index	Not significant
Client satisfaction*	Weak (0.230)
Nonconformance report*	Very weak (0.101)
No. of accidents*	Very weak (−0.114)
Time variance*	Weak (0.263)
Fatalities*	Very weak (−0.111)

*Significant at 0.05 level.

## References

[1] Soetanto R, Proverbs DG, Holt GD (2001). Achieving quality construction projects based on harmonious working relationships—clients' and architects' perceptions of contractor performance. *International Journal of Quality & Reliability Management*.

[2] Xiao H, Proverbs D (2002). The performance of contractors in Japan, the UK and the USA—an evaluation of construction quality. *International Journal of Quality & Reliability Management*.

[3] Kam CW, Tang SL (1997). Development and implementation of quality assurance in public construction works in Singapore and Hong Kong. *International Journal of Quality & Reliability Management*.

[4] Motwani J, Kumar A (1996). A roadmap to implementing ISO, 9000. *International Journal of Quality & Reliability Management*.

[5] Water HVD (2000). A maintenance model for quality management. *International Journal of Quality & Reliability Management*.

[6] Blanchard BS (1986). *Logistic Engineering and Management*.

[7] Gits CW (1984). *On the Maintenance Concept for a Technical System—A Framework for Design. Helmond*.

[8] Torres Peraza M (1995). Planen fur den Tag danach. *Global Quality Management*.

[9] Kim D-Y, Kumar V, Kumar U (2011). A performance realization framework for implementing ISO 9000. *International Journal of Quality & Reliability Management*.

[10] Choi TW, Chin KS (2001). A study of ISO9000 implementation and quality management practices in Hong Kong construction industry. *The Asian Journal on Quality*.

[11] Sharma B, Gadenne D (2002). An inter-industry comparison of quality managament practices and performance. *Managing Service Quality*.

[12] Mo JPT, Chan AMS (1997). Strategy for the successful implementation of ISO 9000 in small and medium manufacturers. *The TQM Magazine*.

[13] Parsa A, Keivani R (1997). Making the most of quality management systems. *Construction Manager*.

[14] Thelen MJ (1997). ISO 9000 and TQM in SITA research and development. *The TQM Magazine*.

[15] Endrijonas J (1994). Certification a bane or a boon?. *Managing Automation*.

[16] Ahmad MFB, Yusof SRM, Yusof NM (2007). Comparative study of quality practices between Japanese and non-Japanese based electrical and electronics companies in Malaysia: a survey. *Jurnal Teknologi*.

[17] Dow D, Samson D, Ford S (1999). Exploding the myth: do all quality management practices contribute to superior quality performance?. *Production and Operations Management*.

[18] Anderson JC, Rungtusanatham M, Schroeder RG, Devaraj S (1995). A path analytic model of a theory of quality management underlying the Deming management method: preliminary empirical findings. *Decision Sciences*.

[19] Jusoh A, Yatim SM (2008). Pelaksanaan ISO, 9000: pengajaran dari kajian baru. *Jurnal Teknologi*.

[20] Ismail MY, El Baradie M, Hashmi MSJ (1998). Quality management in the manufacturing industry: practice vs performance. *Computers and Industrial Engineering*.

[21] Choi TY, Eboch K (1998). The TQM Paradox: relations among TQM practices, plant performance, and customer satisfaction. *Journal of Operations Management*.

[22] Samson D, Terziovski M (1999). The relationship between total quality management practices and operational performance. *Journal of Operations Management*.

[23] Ismail MY, Hashmi MSJ (1999). The state of quality management in the Irish manufacturing industry. *Total Quality Management*.

[24] Adam EE, Corbett LM, Flores BE (1997). An international study of quality improvement approach and firm performance. *International Journal of Operations & Production Management*.

[25] Hendricks KB, Singhal VR (1997). Does implementing an effective TQM program actually improve operating performance? Empirical evidence from firms that have won quality awards. *Management Science*.

[26] Forker LB, Vickery SK, Droge CLM (1996). The contribution of quality to business performance. *International Journal of Operations & Production Management*.

[27] Takim R, Akintoye A, Kelly J (2003). Performance measurement systems in construction. *Association of Researchers in Construction Management*.

[28] Stevens JD (1996). Blueprint for measuring project quality. *Journal of Management in Engineering*.

[29] Sinclair D, Zairi M (1995). Effective process management through performance measurement: part III-an integrated model of total quality-based performance measurement. *Business Process Re-Engineering & Management Journal*.

[30] Mbugua LM, Harris P, Holt GD, Olomolaiye PO A framework for determining critical success factors influencing construction business performance.

[31] Chan A (2001). A quest for better construction quality in Hong Kong. *Construction Information Quarterly*.

[32] Love PED, Holt GD (2000). Construction business performance measurement: the SPM alternative. *Business Project Management Journal*.

[33] Barkley B, Saylor J (1994). *Customer-Driven Project Management*.

[34] Atkinson R (1999). Project management: cost, time and quality, two best guesses and a phenomenon, its time to accept other success criteria. *International Journal of Project Management*.

[35] Kumaraswamy MM, Thorpe A (1996). Systematizing construction project evaluations. *Journal of Management in Engineering*.

[36] Chan APC, Tam CM (2000). Factors affecting the quality of building projects in Hong Kong. *International Journal of Quality & Reliability Management*.

[37] Bubshait AA, Almohawis SA (1994). Evaluating the general conditions of a construction contract. *International Journal of Project Management*.

[38] Salter A, Torbett R (2003). Innovation and performance in engineering design. *Journal of Construction Management and Economics*.

[39] Andi A, Minato T (2003). Design documents quality in the Japanese construction industry: factors influencing and impacts on construction process. *International Journal of Project Management*.

[40] Georgy ME, Chang L-M, Zhang L (2005). Prediction of engineering performance: a neurofuzzy approach. *Journal of Construction Engineering and Management*.

[41] Lim CS, Zain Mohamed M (2000). An exploratory study into recurring construction problems. *International Journal of Project Management*.

[42] Odeh AM, Battaineh HT (2002). Causes of construction delay: traditional contracts. *International Journal of Project Management*.

[43] Latham M (1994). *Constructing the Team*.

[44] Parfitt MK, Sanvido VE (1993). Checklist of critical success factors for building projects. *Journal of Management in Engineering*.

[45] Ganaway NB (2006). *Construction Business Management: A Guide to Contracting for Business Success*.

[46] Locke EA (1970). Job satisfaction and job performance: a theoretical analysis. *Organizational Behavior and Human Performance*.

[47] Ali AS, Rahmat I (2010). The performance measurement of construction projects managed by ISO-certified contractors in Malaysia. *Journal of Retail & Leisure Property*.

[48] Parasuraman A, Zeithaml VA, Berry LL (1988). Service quality: a multi-item scale for measuring consumer perceptions of quality. *Journal of Retailing*.

[49] Soetanto R, Proverbs DG (2004). Intelligent models for predicting levels of client satisfaction. *Journal of Construction Research*.

[50] Sousa S, Teixeira J Prevention measures to reduce risk of falling from heights.

[52] Cochran WG (1977). *Sampling Techniques*.

[53] Sekaran U (2005). *Research Methods for Business: A Skill-Building Approach*.

[54] Deming WE (1982). *Quality, Productivity, and Competitive Position*.

[55] Garvin D (1988). *Managing Quality*.

[56] Steeples MM (1992). *The Corporate Guide to the Malcolm Baldrige National Quality Award*.

[57] Ahmed SM, Aoieong RT, Tang SL, Zheng DXM (2005). A comparison of quality management systems in the construction industries of Hong Kong and the USA. *International Journal of Quality & Reliability Management*.

[58] Low SP, Omar HF (1997). Integration, segmentalism and the maintenance of quality management systems in the construction industry. *Building Research and Information*.

[59] Dick GPM (2000). ISO 9000 certification benefits, reality or myth?. *The TQM Magazine*.

[60] Leung HKN, Chan KCC, Lee TY (1999). Costs and benefits of ISO, 9000 series: a practical study. *International Journal of Quality & Reliability Management*.

[61] Lee TY (1998). The development of ISO, 9000 certification and the future of quality management: a survey of certified firms in Hong Kong. *International Journal of Quality & Reliability Management*.

[62] Chan KW, Chan HC (1997). Meeting quality assurance standards in the construction industry: experience from Hong Kong. *International Journal of Management*.

[63] Tam CM (1996). Benefits and costs of the implementation of ISO, 9000 in the construction industry of Hong Kong. *Journal of Real Estate and Construction*.

[64] Dissanayaka SM, Kumaraswamy MM, Karim K, Marosszeky M (2001). Evaluating outcomes from ISO 9000-certified quality systems of Hong Kong constructors. *Total Quality Management*.

[65] Low SP, Kee TB, Leng AAA (1999). Effectiveness of ISO, 9000 in raising construction quality standards: some empirical evidence using CONQUAS scores. *Structural Survey*.

[66] Zin RM, Chen GH, Ali MC (2009). An observation of impact in implementation of quality management system by contractors. *Malaysian Construction Research Journal*.

[67] Din S, Abd-Hamid Z, Bryde DJ (2011). ISO 9000 certification and construction project performance: the Malaysian experience. *International Journal of Project Management*.

[68] Karim K, Marosszeky M, Kumaraswamy M (2005). Organizational effectiveness model for quality management systems in the Australian construction industry. *Total Quality Management and Business Excellence*.

[69] Seymour D, Low SP (1990). The quality debate. *Construction Management and Economics*.

[70] Low SP (1993). The rationalisation of quality in the construction industry: some empirical findings. *Construction Management and Economics*.

[71] Anonymous (1994). *BS5750/ISO, 9000-Setting Standards for Better Business*.

[72] Brown A, van der Wiele T (1996). A typology of approaches to ISO certification and TQM. *Australian Journal of Management*.

[73] Shammas-Toma M, Seymour DE, Clark L (1996). The effectiveness of formal quality management systems in achieving the required cover in reinforced concrete. *Construction Management and Economics*.

[74] Seymour D (1997). *Assessing Quality Control Systems: Some Methodological Considerations*.

[75] Anderson SW, Daly JD, Johnson MF (1999). Why firms seek ISO 9000 certification: regulatory compliance or competitive advantage?. *Production and Operations Management*.

[76] Moatazed-Keivani R, Ghanbari-Parsa AR, Kagaya S (1999). ISO 9000 standards: perceptions and experiences in the UK construction industry. *Construction Management and Economics*.

